# Need to take action for hypertension: insights from primary care blood pressure practices in Greece

**DOI:** 10.1038/s41371-026-01151-8

**Published:** 2026-04-30

**Authors:** Vasileios Gkolias, Nikolaos Evangelidis, Magda Gavana, Ioannis Staikos, Styliani Ouzouni, Maria Moirasgenti, Anna-Bettina Haidich, Michael Doumas, Emmanouil Smyrnakis, Areti Triantafyllou

**Affiliations:** 1 Laboratory of Primary Health Care, General Practice and Health Services Research, School of Medicine, Aristotle University of Thessaloniki, Thessaloniki, Greece; 2 Primary Health Care Research Network, Aristotle University of Thessaloniki, Thessaloniki, Greece; 3 First Propedeutic Department of Internal Medicine, Hypertension Outpatient Clinic and ESH Excellence Center, AHEPA University Hospital, Aristotle University of Thessaloniki, Thessaloniki, Greece; 4 Department of Hygiene, Social and Preventive Medicine and Medical Statistics, School of Medicine, Faculty of Health Sciences, Aristotle University of Thessaloniki, Thessaloniki, Greece; 5 Second Propedeutic Department of Internal Medicine, Hippokration General Hospital, Aristotle University of Thessaloniki, Thessaloniki, Greece

**Keywords:** Diagnosis, Cardiovascular diseases

## Abstract

Primary healthcare (PHC) physicians play a pivotal role in the diagnosis and management of hypertension. While guidelines suggest that blood pressure (BP) should be measured in every patient visit at PHC units, the few studies conducted among PHC physicians report a low ratio of BP measurements in PHC settings. Data on PHC physicians’ practices concerning BP measurement in Greece are lacking. This study aimed to investigate the practices of PHC physicians regarding BP measurement in Greece. A cross-sectional web-based survey was conducted among PHC physicians across Greece. A total of 284 PHC physicians completed the questionnaire and 282 responses were included in the analysis, 42.9% male, 92.9% General Practitioners, 89.4% working in the public sector, with a median of 25 (17 – 30) daily patient visits. PHC physicians reported measuring BP in 33.3% (20–50%) of their patients and recommending home BP measurements in 31.1% (SD: 24.3%) of them. Among those who measured BP, 22.3% measured it once, 44.7% measured it twice, and only 21.3% measured it three times, while 54.6% used an electronic upper arm BP monitor. The main barriers reported were high daily patient volume (60.5%), together with limited time available with patients (69.5%), while only 1.3% declared insufficient training. This is the first study in Greece investigating the practices of PHC physicians regarding BP measurements. Our findings underscore the need for targeted interventions to improve BP monitoring practices. Education and motivation of physicians and other primary care team members would be fundamental in addressing the challenge of implementing BP measurement recommendations in clinical practice.

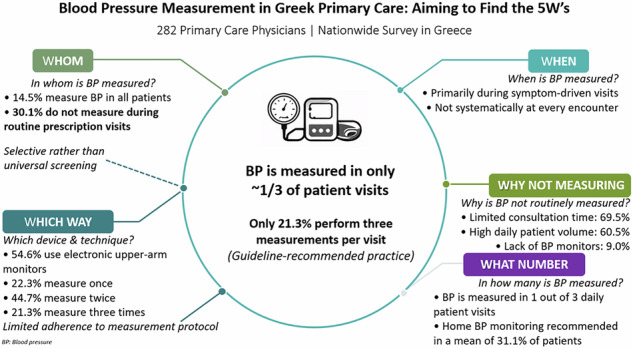

## Introduction

Hypertension (HTN) is the leading cause of avoidable death and disability globally [1], affecting about one-third of the world’s adult population [2]. Nevertheless, an estimated 44% of adults with HTN lack awareness of their condition, and only 23% have their blood pressure (BP) under control [3]. As HTN is often asymptomatic, accurate BP measurement is essential for timely diagnosis and adequate control [4, 5].

Office BP measurements remain the cornerstone of HTN diagnosis, however they carry the risk of often misclassifying patients, due to the considerable proportion of white-coat (30–40%) and masked (~ 13%) HTN in clinical practice [6]. Therefore, out-of-office BP measurements have acquired a pivotal role in HTN diagnosis, classification, and treatment evaluation. Home blood pressure monitoring (HBPM) and ambulatory blood pressure monitoring (ABPM) have been shown to be robust predictors of cardiovascular morbidity and mortality [7]. Furthermore, HBPM actively engages patient cooperation, promoting compliance with antihypertensive therapy [8].

Primary health care (PHC) physicians play a key role in HTN diagnosis and management, as most patients with HTN receive care in PHC settings [9]. The US Preventive Services Task Force (USPSTF) recommends screening for HTN in all adults and confirmation with out-of-office measurements before starting treatment [10]. The European Society of Hypertension (ESH) recommends opportunistic screening for HTN in all adults, and regular BP measurements from the age of 40 years [11]. Nevertheless, current practices of PHC physicians may differ from guideline recommendations [12]. The use of out-of-office BP monitoring remains low [13], while limitations in methodology or device accuracy may lead to over- or under-diagnosis of HTN [14].

The literature on PHC physicians’ practices regarding BP measurement is limited. Relevant studies depict low adherence to current guidelines, with doctors measuring BP only occasionally, usually once per visit, using manual methods or even mercury devices, and not recommending out-of-office measurements [15–21]. To our knowledge, no study has investigated these practices in Greece, data which would be of significant value, since practice behaviors could vary considerably in different countries and health systems. Documenting and understanding these practices are important for identifying and addressing potential gaps in clinical care.

The objective of this study was to survey the practices of PHC physicians regarding BP measurement in Greece, in order to detect deviations from recommendations and bring to light perspective for improvement.

## Materials and methods

### Study design and data collection

A cross-sectional online survey was conducted among board-certified PHC physicians [General Practitioners (GPs), Internists, Cardiologists] employed in public or private PHC across Greece. The survey was anonymous and distributed to physicians through PHC networks, PHC meetings and conferences. Due to this dissemination approach, the total number of physicians reached could not be determined; therefore, the response rate could not be calculated.

Participation in the study was open from November 2024 to October 2025. Data were collected using a 15-item structured questionnaire, which was administered online and developed using Google Forms (Google LLC, Mountain View, CA, USA). The questionnaire was short and required approximately 5 min to complete.

The first section of the questionnaire was designed to gather information on demographic characteristics of the participants (gender, geographical area, and type of healthcare facility of employment, medical specialty, and years since completion of specialty training). The second part of the questionnaire was developed to collect data regarding BP measurement practices, including the number of patients seen during a typical outpatient clinic day, the frequency of office BP measurements, and the use of HBPM. Additionally, this section included questions regarding the type of BP devices used and their validation status, as well as the frequency and indications for BP measurement during routine visits, the number of measurements performed in each patient per visit, and the factors determining the decision whether to measure BP or not. Based on the reported device name, validation status was verified using the STRIDE BP validated device registry [22].

The questionnaire was developed specifically for this study, based on relevant literature and current guideline recommendations. It was designed to collect primarily factual, objective information (e.g., demographic characteristics and frequency-based behaviors). The items were written to be unambiguous to minimize interpretation and reduce measurement error. The questionnaire was not formally validated, and test-retest reliability or inter-rater agreement were not assessed.

Participants were informed about the objectives of the study before participation. Survey completion was voluntary and anonymous, and participants were free to discontinue participation at any stage. Informed consent was obtained from all participants prior to questionnaire completion. All procedures were carried out in accordance with the principles of the Declaration of Helsinki. Ethical approval was obtained from the Institutional Review Board of Aristotle University of Thessaloniki (protocol code: 91/2024; approval date: 16 January 2024).

### Statistical analysis

Statistical analysis was conducted using SPSS 29.0 statistical package (IBM SPSS Statistics for Windows, Version 29.0. IBM Corp, Armonk, NY, USA). Figures were created using R (version 4.3.1). Descriptive statistics of categorical variables are presented as frequencies and percentages (%). Normality of continuous variables was assessed using the Kolmogorov-Smirnov test in combination with visual inspection of histograms and Q-Q plots, as well as evaluation of skewness values. Normally distributed variables are expressed as mean with standard deviation (SD) and non-normally distributed variables as median and interquartile range IQR: the 25th (Q1) and 75th (Q3) percentiles of the distribution were presented.

Comparisons across all the above categories were conducted using the chi-square test for categorical variables, and when expected cell counts were <5, Fisher’s exact test was applied. For continuous variables, the independent samples t-test was used for normally distributed data, while the Mann-Whitney U test was used for non-normally distributed variables.

Logistic regression analyses were performed to investigate which factors were associated with two predefined outcomes: i) Non-routine BP measurement (not routinely measuring BP in patients who come for prescription renewal or routine check-ups) and ii) Failure to perform three BP measurements per patient as recommended by guidelines. Univariate analyses were initially conducted for each independent variable, followed by two separate multivariable regression models, including the same clinically relevant physician characteristics and other variables. Multicollinearity was assessed using the variance inflation factor (VIF), and all variables included in the models had VIF values < 5. A two-sided p-value < 0.05 was considered statistically significant.

## Results

A total of 284 PHC physicians representing all State Health Districts (7/7) in Greece completed the questionnaire. One response was excluded because the participant was a Medical Biopathologist, and another because the participant was a resident and not a consultant. In total, 282 responses were included in the analysis.

**Fig. 1:**
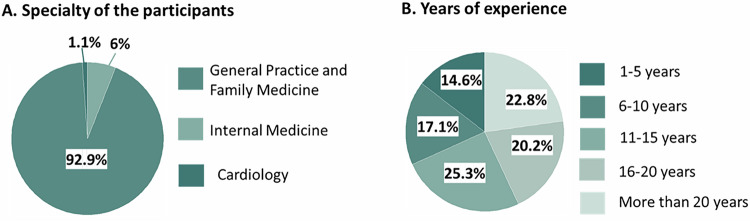
Participant specialty and years since specialty certification. **A** Distribution of participants by medical specialty. **B** Distribution of participants according to years since specialty certification.

Among the participants, 42.9% were male, the vast majority were GPs (92.9%), with 89.4% working in the public sector and 68.3% having >10 years of experience as consultants. Regarding practice setting, 98 (34.8%) worked in urban areas, while 184 (65.2%) practiced in rural areas. Specialty and years of experience are depicted in Fig. 1.

In a median of 25 (IQR 17 – 30) daily patient visits, physicians report measuring BP in a third [33.3% (IQR 20 – 50%)] of their patients, and recommending HBPM in a mean of 31.1% (SD: 24.3%) of them. Regarding the mentioned measurement habits, only 14.5% of physicians measured BP in all patients, while others measured BP only in patients with known HTN (11.4%) or in patients presenting with a problem or symptom requiring clinical examination (44.0%), with less than a third (30.1%) of physicians not routinely measuring BP in patients who come for prescription renewal or routine check-ups. Among all physicians, 22.3% measure it once, 44.7% measure it twice, and only 21.3% measure it three times, while 11.7% state they do not measure BP at all.

**Fig. 2:**
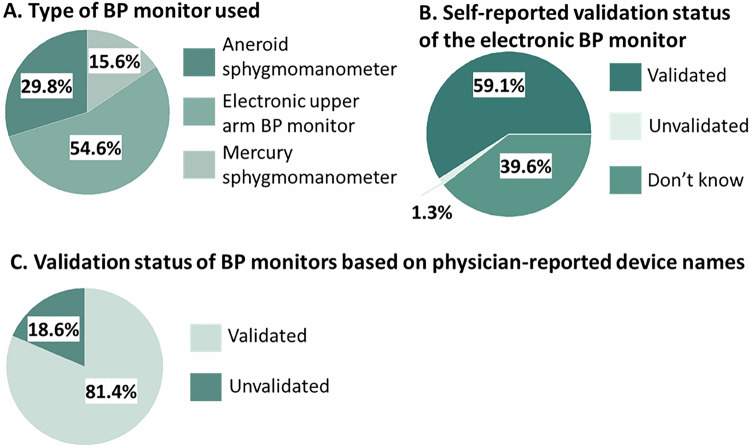
Type and validation status of BP monitors used by physicians. **A** Distribution of types of BP monitors used in clinical practice. **B** Self-reported validation status of the BP monitors used by physicians. **C** Validation status based on the reported device names and verification based on the STRIDE BP validated device registry. *BP:* Blood pressure, *PHC*: Primary healthcare.

Among the responders, most (54.6%) were using an electronic upper-arm BP monitor. Of those, most (59.1%) reported that their BP monitor was validated, while a large proportion (39.6%) were unaware of its validation status (Fig. 2). Nevertheless, among the BP monitors with unknown status, the vast majority (81.4%) were identified by the study investigators as validated.

The physicians who did not use an electronic device stated that the facility they worked in did not have one (22.6%) or that they considered the electronic BP monitor to be less reliable than the mercury or aneroid sphygmomanometer (77.4%).

**Fig. 3:**
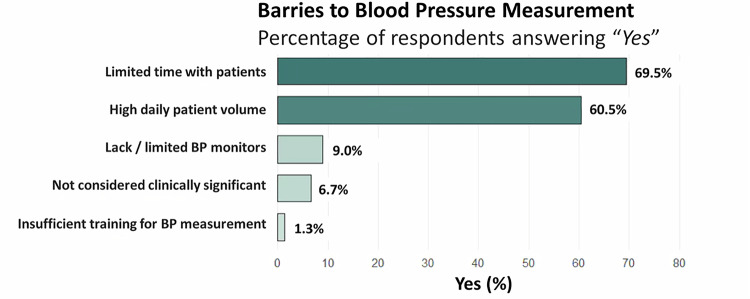
Barriers to BP measurement. *BP:* Blood pressure.

The reported barriers to routine BP measurement are depicted in Fig. 3.

Physicians specialized in Internal Medicine reported measuring BP in a higher proportion of their patients compared to GPs (median 50%, IQR 37.9 – 70.0 vs. 32.1%, IQR 20.0 – 50.0%, p = 0.007). Physicians with fewer than 25 patient visits per day demonstrated a higher office BP measurement rate compared to those seeing ≥25 patients per day (median 40%, IQR 23.5 – 66.7 vs. 28.6%, IQR 16.7 – 40.0%, p < 0.001), while no difference was observed in the rate of recommendation of HBPM [36.5% (SD: 24.7%) vs. 28.3% (SD: 23.7%), p = 0.121].

Half of physicians in the private sector (50.0%) did not routinely measure BP in all patients, compared to 27.8% in the public sector (p = 0.012). Additionally, physicians in the public sector had a higher number of patients per typical clinic day compared to those in the private sector (median 25, IQR 20 – 30.75 vs. 15, IQR 11.5 – 30, p = 0.002).

### Logistic regression analysis

In the multivariable logistic regression model examining non-routine BP measurement according to guidelines, working in the public sector was independently associated with lower odds of not measuring BP (adjusted OR: 0.40, 95% CI 0.18–0.91, p = 0.029). No other physician characteristics were associated with this outcome.

In a separate multivariable model assessing failure to perform three BP measurements per patient according to guidelines, no physician characteristics or other variables were associated with this outcome (Table 1). Results from the univariate logistic regression analyses are presented in Supplementary Table 1.

**Table 1:** Multivariable logistic regression models examining factors associated with (A) non-routine BP measurement and (B) failure to perform three BP measurements according to guidelines.

	Adjusted OR	95% CI	*p*-value
Model A: Factors associated with non-routine BP measurement			
Male gender	1.029	0.620–1.707	0.912
Public sector vs. private sector	0.401	0.176–0.913	0.029
Specialty: GP vs. other specialties	0.896	0.387–2.075	0.798
≥ 20 years of working experience	0.864	0.460–1.623	0.649
Number of patients per day	0.993	0.969–1.019	0.601
Use of electronic BP monitor	1.216	0.719–2.059	0.465
Model B: Factors associated with not performing three BP measurements			
Male gender	1.436	0.810–2.546	0.216
Public sector vs. private sector	0.542	0.202–1.451	0.223
Specialty: GP vs. other specialties	2.693	0.742–9.779	0.132
≥ 20 years of working experience	1.360	0.695–2.661	0.369
Number of patients per day	0.998	0.971–1.027	0.914
Use of electronic BP monitor	1.413	0.772–2.585	0.262

Two separate multivariable logistic regression models were conducted. In Model A, the dependent variable was non-routine BP measurement (not routinely measuring BP in patients who come for prescription renewal or routine check-ups). In Model B, the dependent variable was failure to perform three BP measurements in each patient as suggested by guidelines.

*BP* blood pressure, *CI* confidence interval, *GP* general practice, *OR* odds ratio.

## Discussion

The ESH recommends opportunistic screening for HTN in all adults, emphasizing the importance of regular BP measurements, at least in those over the age of 40 and in those at increased risk for HTN, further recommending BP measurements during medical or prescription-related visits [11]. Nevertheless, this concept has not been incorporated into clinical practice, as shown in our study, conducted in PHC, since physicians report measuring BP in only 33.3% of the patients they see in a typical day at the office. Only 14.5% of them claim that they measure BP in all patients at every visit, while 11.7% report they don’t measure BP at all. Our study also revealed that only 21.3% of the study participants measure BP three times per visit, whereas only a mean of 31.1% stated that they ask their patients to provide home BP measurements. The main reasons reported for not measuring BP were high daily patient volume, together with limited time available with patients, a deduction further supported by the negative effect of the number of daily patient visits on BP measurement rates.

This gap in the implementation of current guidelines in everyday clinical practice does not come as a surprise. In German medical practices nationwide, only a single BP measurement was performed in 66.3% of the cases, whereas 62.3% of patients with known HTN recorded home BP measurements [15]. Similarly, HBPM before establishing a final diagnosis was (usually or always) recommended in 67% of their patients by family physicians in Spain [23]. In a study in the United Kingdom, 65.8% of patients reported having their BP measured during their last clinic visit, whereas only one BP reading was obtained in 59.6% of them [19]. It has been estimated that GPs, internists and cardiologists working in primary care and outpatient clinics in Hungary measure office BP in less than one tenth of their patients [24]. Physicians working in three kinds of health institutions (mainly primary care, but also secondary and tertiary care) in Turkey were found to measure BP in 37% of their patients, only once per patient in all cases, while 17.9% of them never measured the patients’ BP [20]. Office BP estimation was based on the mean of two measurements in 58.5% of PHC clinics in Utah, USA [21]. In a study in Hong Kong, 26.7% of primary care doctors report obtaining only one BP reading for the diagnosis and/or treatment of HTN, while 22.2% use HBPM for establishing the diagnosis and 56.8% to guide the management of HTN [17]. In a qualitative study in Washington, USA, PHC physicians preferred office BP measurement for the diagnosis of HTN, as more accurate, provided optimal conditions were met. Concerning HBPM, they feel that patients may be unable to follow recommended practices, may use unreliable devices, and that the process would cause patient anxiety [25]. In a survey in PHC practices in South West England, on the occasion of a BP reading being above 140/90 mmHg, 33% of practices state that they would obtain further office measurements, while 30% would recommend ABPM, 44% HBPM and 26% would offer either choice [18].

The challenges faced by physicians, contributing to suboptimal guideline adoption regarding BP measurement practices, seem universal. Principal barriers worldwide in achieving acceptable levels of HTN detection and control include lack of effective PHC, clinical inertia and low physician-to-patient ratio [26]. Physicians face several constraints in implementing ABPM and HBPM, ranging from context and resources to reliability of acquired data [27]. PHC physicians in the USA state that they do not trust HBPM data, citing concerns about the accuracy of the devices used and suboptimal measuring conditions [25].

In our study, PHC physicians working in the private sector reported lower rates of BP measurement in their patients, compared to those working in the public sector, despite having a lower number of patients per average office day. We assumed that this difference might be explained by the fact that nurses are assigned to public primary care doctors’ offices, undertaking this duty, which is quite common in Greek public primary care facilities, a fact that does not apply to private primary physicians’ practices. However, this data was not specifically obtained in our study. The high number of daily patient visits ( ≥ 25) had a negative effect on office BP measurement rates, yet this is a structural parameter in the health system, not easily modifiable.

In our study, 54.6% of physicians used electronic upper arm BP monitors, 29.8% aneroid (analog) and 15.6% mercury sphygmomanometers, with those not using electronic devices stating either that such a device is not available in the healthcare facility they work in or that they don’t consider it as reliable as aneroid or mercury sphygmomanometers. Similarly, Washington PHC physicians were in favor of manual BP measurement, stating that this is what healthcare professionals are trained to do well [25]. Family physicians in Canada prefer manual BP measurements with a mercury or aneroid device for screening for high BP (54.2%), followed usually by automated office BP measurements (31.1%) and less frequently by HBPM (22.4%) or ABPM (14.4%) to confirm the diagnosis [16]. The majority of PHC physicians in Hong Kong reportedly use manual BP measurement methods, using aneroid or even mercury devices [17]. Surprisingly, and contrary to studies demonstrating that manual BP measurements are prone to error [28, 29], healthcare professionals in the USA tend to believe that manual BP measurement with aneroid sphygmomanometers are more accurate than automated BP measurements [30]. Recorded office BP measurements can be inaccurate, if not carried out according to practice recommendations, regarding both the methods and the devices used. Inaccurate recording of office BP can lead to falsely high BP values [31]. Misdiagnosis based on office BP measurements alone leads to labelling people as hypertensive or prehypertensive, subsequent unnecessary treatment and avoidable health care costs [32].

### Strengths and limitations

This is one of the few studies investigating physician practices regarding BP measurement and the first one in Greece. The study was conducted across the entire country, in all State Health Districts, among physicians representing a wide range of professional experience. Finally, our study took place after the COVID-19 pandemic and would therefore be depicting physician practices that may have been evolving, due to a shift in focus towards out-of-office care.

The findings of this study should be interpreted with caution. Our online survey was based on convenience mechanisms for recruitment, therefore it is prone to selection bias, as the physicians who completed the questionnaire might have more interest and experience in HTN care. As with most self-reported data, there is a possibility of social desirability bias, with responders selecting answers that are considered more appropriate, but do not necessarily reflect actual practices, which might be skewing our results towards more acceptable practices. Nevertheless, this would make our findings of physician practices deviating from guideline recommendations even more robust. Most of the participating physicians were employed in the public sector, where conditions and practices might differ from those in the private sector.

Our study did not evaluate whether physicians interpreted repeated BP measurements correctly, i.e. calculating the average of the last two readings when three measurements were obtained, as recommended by current guidelines [11]. The predominance of GPs and the limited representation of other specialties (such as internists and cardiologists) restricted comparisons across specialties and may have reduced the discriminatory power of the regression analyses. Finally, the total number of physicians who received the invitation to participate could not be determined, due to the way in which the survey was disseminated; therefore the response rate could not be calculated, limiting the assessment of the representativeness of the study population.

### Perspectives

It is a fact that HTN prevalence has increased due to high salt intake, sedentary lifestyle, and high rates of obesity. While HTN remains undiagnosed and uncontrolled at unacceptable rates, it is apparent that improvement in these domains, through implementation of appropriate strategies, is achievable [33]. Physicians in our study didn’t identify inadequate training as a barrier in routine BP measurement in everyday practice, yet their clinical skills and guideline awareness were not actually verified. Hence, tangible interventions applicable in the majority of PHC settings are essential. In this direction, an option would be to recruit more health professionals, beyond primary care physicians, to take action in this field. Practice nurses working in PHC units, as well as health care assistants, have been increasingly capable and active in BP monitoring, aiding physicians in HTN diagnosis and management [18, 30]. Recently, we showed that in Aristotle University of Thessaloniki, Greece, the active involvement of final-year medical students has contributed to the identification of undiagnosed and uncontrolled patients with HTN [34]. Further research in this setting, with carefully designed interventions studied prospectively, would validate the applicability and establish the effectiveness of strategies addressing the challenge of implementing BP measurement recommendations in everyday clinical practice. Additionally, future studies should further investigate how ABPM is performed in PHC and explore physicians’ knowledge and attitudes toward its use. Future research should focus on the development of a study specifically designed to include a large number of physicians from both public and private primary care settings, across different specialties, enabling a comprehensive and comparative understanding of BP measurement practices across healthcare sectors in Greece.

## Conclusion

There is an urgent need to motivate and educate physicians and other PHC team members on best practices, providing incentives rewarding HTN prevention, screening and control. Re-organizing PHC framework, taking advantage of modern technologies and artificial intelligence and promoting protocolized, team-based care could be instrumental in obtaining reliable data, tackling therapeutic inertia and engaging clinicians and patients in a productive continuity in HTN care.

## Summary

### What is known about the topic

- Accurate blood pressure measurements are key for HTN detection, diagnosis, control and follow-up.
- Current practices of PHC physicians may differ from guideline recommendations.
- The literature on PHC physicians’ practices regarding BP measurement is limited.

### What this study adds

- Physicians in Greece measure office BP in a low proportion of their patients.
- Only one-out-of-five measure BP three times per visit, with approximately half of them using electronic upper arm BP monitors.
- They recommend HBPM in about one-third of their patients.
- The main barriers reported are high daily patient volume, together with limited time available with patients.

## Supplementary information

Supplementary Table 1. Univariate Logistic Regression Analysis

## Data Availability

The data that support the findings of this study are available from the corresponding author upon request.
