# Supplementary material for: Need to take action for hypertension: insights from primary care blood pressure practices in Greece
Source: J Hum Hypertens. 2026 Apr 30;40(6):489–94. doi: 10.1038/s41371-026-01151-8 (PMC13249564; doi:10.1038/s41371-026-01151-8)
Supplement: Supplementary file 1 — Supplementary Table 1. Univariate Logistic Regression Analysis [file 41371_2026_1151_MOESM1_ESM.docx]

Supplementary Table 1. Univariate Logistic Regression Analysis

|  | **OR** | **95% CI** | **p-value** |
| --- | --- | --- | --- |
| **Univariate logistic regression analysis of factors associated with non-routine BP measurement** | | | |
| Male gender | 1.095 | 0.668 -1.796 | 0.718 |
| Private sector | 0.385 | 0.179 -0.828 | 0.015 |
| Specialty: general practice | 0.713 | 0.329 -1.545 | 0.391 |
| ≥ 20 years of working experience | 0.857 | 0.464 -1.586 | 0.624 |
| Number of patients per day | 0.999 | 0.975 -1.024 | 0.929 |
| Use of an electronic BP monitor | 1.277 | 0.764 -2.137 | 0.351 |
| Use of a certified BP monitor | 1.275 | 0.490 -3.317 | 0.618 |
| Measure BP 3 times, according -the guidelines | 1.003 | 0.505 -1.993 | 0.993 |
| **Univariate logistic regression analysis of factors associated with not performing three BP measurements** | | | |
| Male gender | 1.442 | 0.820 -2.534 | 0.204 |
| Public sector | 0.684 | 0.281 -1.664 | 0.403 |
| Specialty: general practice | 1.928 | 0.609 -6.100 | 0.264 |
| ≥ 20 years of working experience | 1.265 | 0.658 -2.432 | 0.481 |
| Number of patients per day | 1.001 | 0.974 -1.029 | 0.949 |
| Percentage of patients with BP measured | 1.001 | 0.989 -1.013 | 0.869 |
| Use of an electronic BP monitor | 1.494 | 0.825 -2.704 | 0.185 |
| Use of a certified BP monitor | 1.684 | 0.522 -5.431 | 0.383 |

*BP*: Blood pressure, *CI*: Confidence interval, *OR*: Odds ratio
